# Evaluating cardiovascular risk in metabolic steatosis with precision medicine non-invasive approaches: insights from a cohort study

**DOI:** 10.1007/s11739-024-03626-3

**Published:** 2024-05-16

**Authors:** Mario Masarone, Benedetta Maria Motta, Pietro Torre, Marco Aquino, Federica Belladonna, Martina Lombardi, Jacopo Troisi, Marcello Persico

**Affiliations:** 1 Department of Medicine, Surgery and Dentistry, “Scuola Medica Salernitana”, University of Salerno, Baronissi, SA Italy; 2 Theoreo srl, Montecorvino Pugliano, SA Italy

**Keywords:** MASLD, NAFLD, Cardiovascular risk, Metabolomics, Single nucleotide polymorphisms

## Abstract

Metabolic associated steatotic liver disease (MASLD) is the most common liver condition. It is associated with increased liver-related morbidity and mortality, and also with high risk of cardiovascular events (CVD), representing itself an independent risk factor for it. This makes MASLD a presentation of high interest for internal medicine, also because of its association with metabolic syndrome (MetS). It is crucial to assess its risks in a noninvasive way. With the aim of finding specific risk profiles for CVD development in MASLD by performing a noninvasive assessment of: (1) preclinical signs of endothelial dysfunction (ED); (2) clinical assessment of CVD risk by Framingham Heart Risk Score (FHRs); (3) genomic characterization of MASLD associated polymorphisms; (4) specific untargeted metabolomic profiles, we enrolled 466 MASLD patients non-invasively classified in 4 group of liver fibrosis severity (group-A: low-fibrosis risk, group-B: high-fibrosis risk, group-C: MASLD-cirrhosis, group-D: MASLD-HCC) and 73 healthy controls. FHRs was similar in controls and low-fibrosis group and significantly higher in high-fibrosis patients, cirrhosis, and HCC, increasing among classes. At a multivariable regression, FHRs was associated with liver disease severity and diabetes. 38.2% of patients had altered EndoPAT, resembling ED. Patients with high FHRs (> 40%) and ED had different metabolomics compared to those without ED. Our study reveals that a deep, non-invasive characterization of MASLD patients through precision medicine approaches (untargeted metabolomics, SNPs, ED assessment) was able to show a peculiar pattern in MASLD patients with increased CVD risk, mostly correlated with liver disease severity.

**Graphical abstract:**

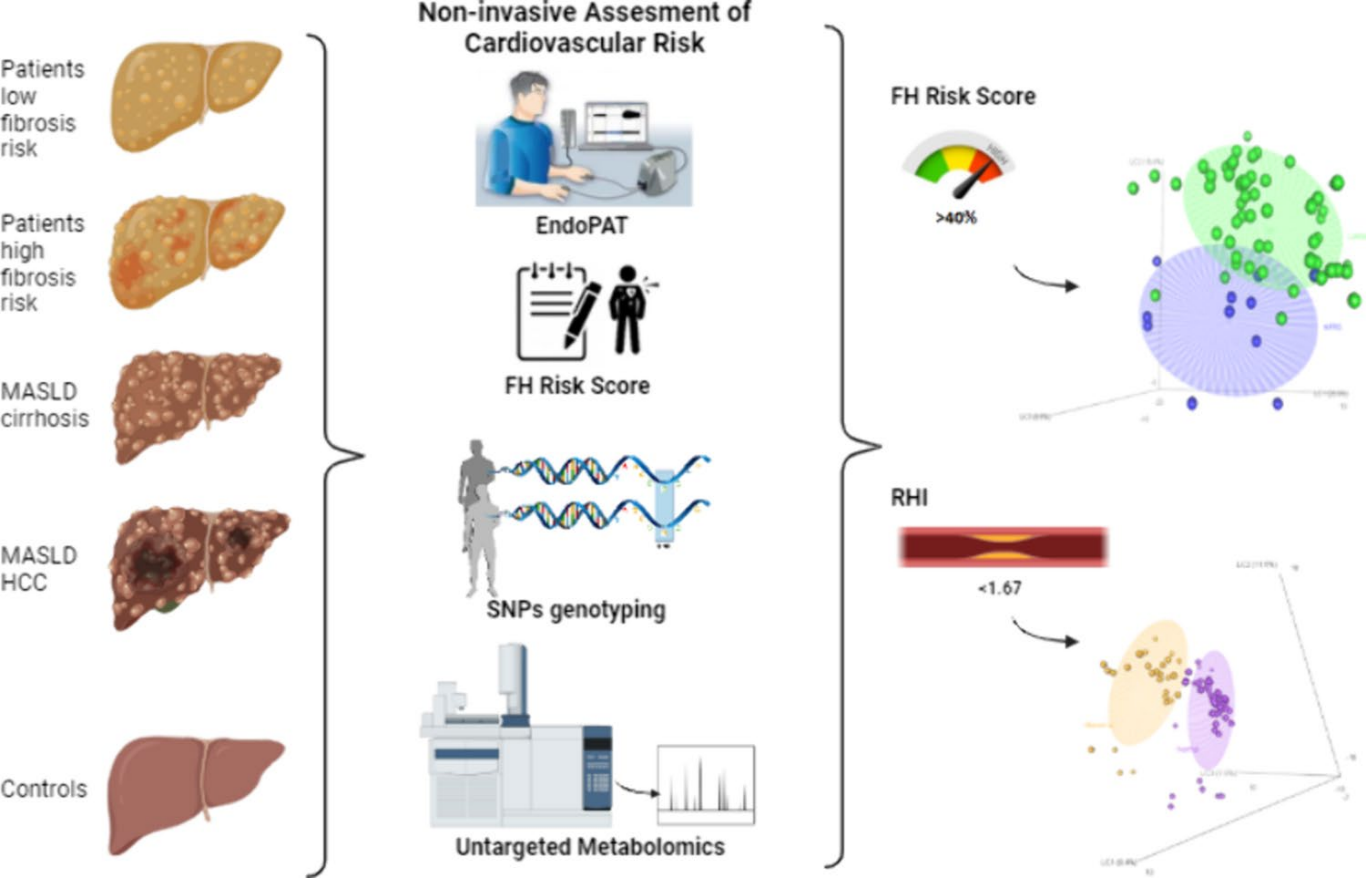

**Supplementary Information:**

The online version contains supplementary material available at 10.1007/s11739-024-03626-3.

## Introduction

Metabolic steatosis, formerly known as nonalcoholic fatty liver disease (NAFLD), defined as a significant accumulation of fat in the hepatocytes (more than 5%) in those who do not consume unsafe quantities of alcohol, is the emergent liver disease. It is estimated that its prevalence is about 20–30% of general population in western countries and 5–18% in Asia, and even more represented in patients with obesity (54–90% among the studies) and/or metabolic syndrome (78.8%) [1, 2]. It is rapidly increasing, especially in developing countries, due to the spread of Western lifestyle and its associated conditions: sedentary lifestyle, obesity, dyslipidemia, insulin resistance, metabolic syndrome and diabetes [3]. Due to the strict connection with these clinical aspects, a consensus of experts firstly promoted a change in its nomenclature to metabolic-associated fatty liver disease (MAFLD) [4], then, recently, a multi-society Delphi consensus statement proposed to further changing the definition to metabolic dysfunction-associated steatotic liver disease (MASLD) [5]. These recent frequent changes of terminology, and the associated debate in the scientific community, highlight how important are the pathogenetic features of this condition in its natural history. As already mentioned, those features are mostly represented by obesity, insulin resistance, type 2 diabetes mellitus (T2DM) and endothelial dysfunction, which are in common with the cardiovascular diseases (CVD) ones, such as angina, myocardial infarction, and stroke [6]. Evidence of this stringent pathophysiological association is that CVD is the most common cause of morbidity and mortality in patients with MASLD, making it an independent CVD risk factor, and even promoting the development of CVD, hypertension and T2DM [6, 7].

Therefore, MASLD patients have an increased overall mortality in comparison to matched control patients, which is only partially due to liver-related deaths [8]. This peculiar pathophysiology makes this disease a topic of high interest for Internal Medicine specialty, due to its multi-organic involvement [9]. Since the already high global burden of MASLD continue to increase, internal medicine physicians will very likely more and more face the difficulty, in their everyday clinical practice, to identify those patients who are at higher risk, and also which type of risk (liver- or CVD-related) they have [10]. To perform this task is of crucial importance in a screening setting; indeed, many efforts have been made to identify a way to perform clinical evaluation with non-invasive methods that could be both reliable and applicable to a large number of patients. In fact, a non-invasive approach has been indicated as the preferred way to identify patients at highest risk of advanced liver disease, as well as of progression and mortality [11]. As a matter of fact, nowadays, all the most updated scientific societies guidelines on the management of NAFLD consider non-invasive methods to assess its severity [12]. In example, the Italian guidelines on NAFLD, promoted by the Italian Association for the Study of the Liver (Associazione Italiana Studio Fegato—AISF), indicated that, in NAFLD patients, a two-tier sequential combination of noninvasive scores (Fibrosis-4 score—FIB-4 and NAFLD fibrosis score—NFS), and liver stiffness measurement (LSM) by transient elastography, have acceptable accuracy to identify those cases at low risk of advanced fibrosis, thus allowing to identify subjects at high risk of advanced fibrosis, for further assessment [2]. Even if this method has been demonstrated to have good supporting evidence, it is mostly limited to liver disease assessment (namely fibrosis), and only by inference to other complications, such as CVD risk.

The Framingham Hearth Risk Score (FHRs) was firstly developed in 1998 on the basis of data coming from the Framingham Heart Study, to estimate the 10-year risk of developing coronary heart disease [13]. Subsequently, in order to better assess the cardiovascular risk, on top of coronary heart disease, also cerebrovascular events, peripheral artery disease and heart failure were added as disease outcomes and the 2008 Framingham Risk Score was developed [14]. Although the questionable impact of such CVD risk scores in patients’ clinical outcomes, FHRs can reliably individuate those at higher risk of CVD events at 10 years, and may direct clinicians’ decisions about prevention and treatment of the higher risk subjects [15]. In the 2008 version, it indicates that individuals have a risk at 10 years that is low when it is 10% or less, intermediate when it is 10–20%, and high when it is 20% or more. However, it should be pointed out that these categories have been arbitrarily selected.

Digital peripheral artery tonometry (PAT) is a novel noninvasive method to assess endothelial and microvascular dysfunction by measuring reactive hyperemia in the blood micro-vessels of the fingers. It has been reported that it is correlated with CVD risk and the presence of coronary artery disease, also at a preclinical stage [16, 17]. Recently, it is also been related to cardiovascular events in a follow-up study carried out in the Framingham cohort [18].

In the picture of its pathophysiological assessment, a high interest is posed on the genetics of NAFLD. Several genome-wide association and candidate gene studies have identified single nucleotide polymorphisms (SNP) that have been associated with NAFLD onset, severity, and peculiar clinical presentations. Among these, I148M *PNPLA3* variant is recognized as the most common genetic determinant of NAFLD onset and progression towards Nonalcoholic steatohepatitis (NASH) fibrosis and even hepatocellular carcinoma (HCC) [19, 20]. Other variants with moderate effect size (but with peculiar manifestations) have been also reported in other genes, such as in *TM6SF2*, *MBOAT7* and *GCKR* [21]. Transmembrane 6 superfamily member 2 (*TM6SF2*) is involved in the secretion of very low-density lipoproteins from the hepatocytes. Its rs58542926 C>T polymorphism seems to confer a higher risk of liver disease but lower risk of CVD events [22]. Membrane bound O-acyltransferase domain-containing 7 (*MBOAT7*) locus rs641738 C>T variant has been associated with reduced levels of phosphatidyl-inositol containing arachidonic acid in hepatocytes and in the circulation, leading to higher risk of NAFLD, inflammation, fibrosis and HCC [23, 24]. The glucokinase regulator (*GCKR*) gene variant rs1260326 has been associated with hepatic fat accumulation via the dysregulation of glucokinase, thereby activating glucose uptake and lipogenesis in the liver [25].

Altogether, these SNPs are closely related to lipid metabolism derangement in the liver and at the systemic level.

Untargeted metabolomics is a powerful novel method to study diseases pathophysiology by mean of a comprehensive analysis of intermediate and end products of various biochemical pathways. It has the advantage to evaluate more accurately the “phenotype” of a disease, in respect to genes, transcripts and proteins, which very likely undergo to epigenetic, transcriptional and pre-/post-translational modifications [26]. Our group recently reported how untargeted metabolomics, analyzed with a GC–MS technique, was able to discriminate among the various stages of NAFLD [27]. By applying this technique on MASLD patients characterized by non-invasive tools in respect of their liver disease severity and their CVD risk, it could be possible to individuate peculiar metabolic phenotypical patterns able to discriminate those at high risk of CVD.

## Aim

The aim of the present study is to deeply characterize patients with MASLD, by mean of pre-clinical, clinical, genetics and metabolomics study in respect to their clinical risk of CVD, in the most non-invasive way possible, to offer an insight into patient management in daily clinical practice.

## Patients and methods

### Patients and controls

Four hundreds and sixty-six patients with ultrasonographical evidence of liver steatosis (bright liver echo pattern) coming as outpatients in a hepatology tertiary center of southern Italy, were consecutively enrolled from January 2018 to December 2022 to participate in this single center observational study. At the time of the enrolling, the exclusion criteria comprehended any unsafe alcohol consumption in the personal history, any other cause of liver disease (autoimmune, viral, metabolic other than NAFLD) and also the absence of any of the metabolic syndrome components (obesity, hyperglycemia or diabetes, hypertension, hypertriglyceridemia, low HDL cholesterol). Therefore, this method of selection, aimed at building up a cohort of NAFLD patients with metabolic derangements with the scope of precisely investigate the CVD risk of associated steatosis allowed us to define them as MASLD patients, after the introduction of the new nomenclature. Also 73 age- and sex-matched healthy subjects were recruited by a local blood bank as controls.

### Clinical evaluation

For each subject, we recorded: clinical history, physical examination, biochemical data, a complete drug history. We performed liver disease assessment and stratified the patients in three categories: low risk of advanced fibrosis [group A: subjects with a NFS lower than − 1.455 and a FIB-4 < 1.30 or NFS > − 1.455 and/or FIB-4 > 1.30 and a LSM < 8 kPa or no significant fibrosis at the liver biopsy (F0–F1)], high risk of advanced fibrosis [group B: patients with a FIB-4 > 1.30 and/or a NFS > − 1.455 and a LSM > 8 kPa or significant fibrosis at the liver biopsy (F2–F3)], and clinical cirrhosis (group C). A group of patients with a diagnosis of steatosis-associated hepatocellular carcinoma were also included (group D). More details in Supplementary data.

### Framingham Heart Risk Score calculation

Age, sex, smoking habit, systolic and diastolic blood pressure, total cholesterol, low-density lipoprotein (LDL) cholesterol, high-density lipoprotein (HDL) cholesterol, and triglycerides levels were collected for the evaluation of CVD risk at 10 years by the Framingham Heart Risk (FHR) score calculation as by European Guidelines indications [14, 28]. More details in Supplementary data.

### EndoPAT evaluation

To evaluate the presence of endothelial dysfunction, which is the preclinical sign of CVD, finger plethysmography (Endopat2000, Itamar) was performed in a subset of patients, randomly selected. This technique measures pulsatile arterial volume changes in fingers by means of plethysmographic probes before and after reactive hyperemia induced by occluding blood flow through the brachial artery for 5 min using an inflatable cuff on one hand. A reactive hyperemia index (RHI) below 1.67 was defined as endothelial dysfunction, in line with the manufacturer’s recommendations [29].

#### Serum and plasma collection

Serum and EDTA plasma collection tubes were provided by BD Vacutainer^®^ (Becton Dickinson Italia S.p.A). Serum and plasma aliquots were recovered from patients’ and controls’ samples collected after an overnight fasting and transferred into prelabeled cryovials and store at − 80 °C (Supplementary data).

### Genetic study

#### SNPs genotyping

DNAs have been extracted from peripheral blood. The rs738409 (I148M, *PNPLA3*), rs58542926 (E167K, *TM6SF2*), rs641738 (*MBOAT7*), and rs1260326 (P446L, *GCKR*) SNPs have been genotyped by TaqMan 5’-nuclease allelic discrimination assays.

The contribution of genetic factors was estimated by assuming an additive, dominant or recessive genetic model, separately (Supplementary data).

### Metabolomics analysis

Metabolomics evaluation was performed by mean of a GC–MS analysis; the extended methodology has been already published [27, 30, 31]. In brief, the metabolome extraction, purification, and derivatization were conducted using the MetaboPrep GC kit (Theoreo, Montecorvino Pugliano, Italy) according to the manufacturer’s instructions. 2-isopropyl malic acid was used as the internal standard. Instrumental analyses were performed with a GC–MS system (GC-2010 Plus gas chromatograph and QP2010SE mass spectrometer; Shimadzu Corp., Kyoto, Japan). The analytical details are reported in Masarone et al. [27]. More details in Supplementary data.

### Statistical analysis

#### Clinical and genetics data

Data are reported as mean ± standard deviation for continuous variables and number (percentage) for categorical variables. Statistical analysis was performed using IBM SPSS Statistics for MacIntosh, Version 26.0 (IBM Corp. Armonk, NY, Released 2019). Normal distribution of data was verified using the Shapiro–Wilks test. Since the data were normally distributed, we used one-way ANOVA with the Tukey post hoc test for inter-group comparisons. Pearson’s Chi squared test was used to determine differences among groups for the categorical variables. Multivariate analyses were performed by mean of a multiple linear regression when the dependent variable was continuous and a multiple logistic regression when the dependent variable was dichotomous. The alpha (*α*) value was set to 0.05 in a two-tails comparison.

### Metabolomics data

#### Dataset preparation

Within each total ion count (TIC) chromatogram, > 300 signal peaks were detected in each specimen. Chromatograms were first aligned by means of parametric time warping (PTW) using the PTW package [32]. Some of the peaks were not investigated further, as they were not consistently found in at least 80% of the samples, were too low in concentration, or were of poor spectral quality to be confirmed as metabolites. A total of 242 endogenous metabolites were detected consistently. The aligned chromatograms were tabulated with one sample per row and one metabolite area ratio (with respect to the internal standard area) per column. Each value was transformed by taking the natural log and then scaled by mean-centering and dividing by the standard deviation of that column (i.e., autoscaled) [33].

#### Feature selection

To reduce the dataset dimension and focus the analysis on the most relevant metabolites, a process referred to as feature selection was performed using a genetic algorithm that is a heuristic search that mimics the process of natural evolution such as inheritance, mutation, selection, and crossover [34] (Supplementary data).

#### Partial least square discriminant analysis (PLS-DA)

PLS-DA was performed to find the combination of metabolites that best separated the different classes on the basis of a specific metabolomic profile (Supplementary data). A permutation test was performed to verify the significance of class discrimination. For each permutation, a PLS-DA model was built between the data and the permuted class labels using the optimal number of components determined by cross-validation for the model based on the original class assignment. Two types of test statistics were used to measure class discrimination. The first was based on prediction accuracy during training. The second made use of separation distance based on the between/within distance ratio (B/W). If the observed test statistics was part of the distribution based on the permuted class assignments, class discrimination could not be considered significant from a statistical point of view [35].

The “Metacost” algorithm was used to correct the imbalance effect for each class [36].

#### Identification of relevant metabolites

Two separate selection strategies were used to find the most relevant metabolites. First, the importance of each metabolite in class separation was evaluated using the variable importance in projection (VIP) scores [37] calculated for each metabolite used in the PLS-DA classification model. Second, metabolites were selected based on their fold change (FC) and *t* test-based *p* values (Volcano plot). Metabolites that showed both FC > 2 or FC < − 2 and *p* values lower than 0.05 were selected (Supplementary data).

## Results

Of the 466 patients included, after the clinical work-up, which included (as described in the methods section) the non-invasive assessment of liver disease severity by mean of the diagnostic algorithm proposed by AISF [2], 227 subjects were defined to have a “steatosis with low risk of significant fibrosis” (group A). One hundred and one patients were defined to have steatosis with “high risk of significant fibrosis” (group B), see methods section for more information on the groups’ classification.

One hundred and five patients with NAFLD-associated clinical cirrhosis (group C) and 33 NAFLD-associated HCC (group D) were also enrolled. The demographical data of the study population are summarized in Table 1. Overall, MASLD population (*n*. 466) compared to age- (66.71 vs 65.35 years; *p*: ns) and sex-matched (male sex 58.1 vs 51.38%; *p*: ns) controls (*n*. 73) presents higher mean BMI (30.52 vs 23.32 kg/m^2^; *p*: 0.021), GGT (83.60 vs 55.96 U/L; *p*: 0.025), glycaemia (116.85 vs 96.77 mg/dL; *p* < 0.0001), LSM (13.21 vs 5.08 kPa; *p* < 0.0001), FIB-4 (3.29 vs 2.81; *p*: 0.018), higher rates of diabetes (46.4 vs 13.84%; *p* < 0.0001), hypertension (72.0 vs 60.93%; *p*: 0.039), metabolic syndrome (41.3 vs 13.2%; *p* < 0.0001) and *PNPLA3* dominant genetic profile (CG + GG) (56.8 vs 37.93%; *p*: 0.035). No significant differences were found in AST, ALT, Total and HDL cholesterol, triglycerides, and for the other genetic profiles (*MBOAT* recessive, *GCKR* recessive, *TM6SF2* dominant). When comparing the single patients’ groups, patients in group A compared to controls, had significantly higher mean BMI (31.34 vs 23.32 kg/m^2^; *p* < 0.0001), glycaemia (116.18 vs 96.77 mg/dL; *p*: 0.001), total cholesterol (173.21 vs 152.45 mg/dL; *p*: 0.004), higher rates of diabetes (39.16 vs 13.84%; *p* < 0.0001), metabolic syndrome (47.05 vs 13.20%, *p* < 0.0001) and a lower FIB-4 (1.56 vs 2.81; *p* < 0.001) but with equal LSM (7.76 vs 5.08, *p*: ns). Moreover, those with high risk of fibrosis (group B), compared to group A had significantly higher mean ALT (53.10 vs 41.05 U/L; *p*: 0.039), GGT (89.82 vs 58.61; *p*: 0.025), FIB-4 (3.71 vs 1.56; *p* < 0.0001), LSM (13.629 vs 7.76; *p* < 0.0001), FHR score (35.81 vs 23.28; *p*: 0.004), a higher rate of PNPLA dominant genetic profile (71.42 vs 50.6%; *p*: 0.019). Patients of group B compared with NAFLD patients with clinical cirrhosis (group C) differ only for higher levels of triglycerides (156.42 vs 103.73 mg/dL; *p* < 0.0001) and lower values of FIB-4 (3.71 vs 5.30, *p*: 0.20) and LSM (13.629 vs 25.23 kPa; *p*: 0.003). In the same way, patients of group C (NAFLD-related clinical cirrhosis) differ from group D (NAFLD-HCC) only for lower mean GGT (122.48 vs 224.29 U/L; *p*: 0.011) FHR (31.90 vs 49.47%; *p*: 0.044) and LSM (25.23 vs 54.07 kPa; *p*: 0.0139). Moreover, also an analysis of the medications that might affect metabolism and CVD (lipid-lowering agents, hypertension and antidiabetic drugs) was performed and, being the results consistent with the prevalence of the corresponding conditions, were reported as supplementary Table 1 (see supplementary material).

**Table 1:** Demographical data of the study population

	Overall (466)	<*p*>	Controls (73)	<*p*>	MASLD (466)							*p* overall
					Group A (227)	<*p*>	Group B (101)	<*p*>	Group C (105)	<*p*>	Group D (33)	
Age (years). mean	66.71	0.073	65.35	0.322	62.81	0.390	70.01	0.748	69.35	***0.054***	74.65	***0.000***
Male sex (%)	58.15	0.175	51.38	0.411	57.26	0.108	67.32	0.455	58.09	0.377	69.69	0.337
BMI (kg/m^2^). mean	30.52	***0.021***	23.32	***0.000***	31.34	0.967	31.25	0.382	32.76	0.536	31.24	0.786
AST (U/L). mean	43.36	0.545	42.24	0.300	35.05	0.683	44.41	0.286	50.38	0.057	76.29	***0.002***
ALT (U/L). mean	44.05	0.603	43.52	0.751	41.05	***0.039***	53.10	0.146	42.02	0.488	47.53	0.358
GGT (U/L). mean	83.60	***0.025***	55.96	0.813	58.61	***0.025***	89.82	0.084	122.48	***0.011***	224.29	***0.000***
Glycemia (mg/dL). mean	116.85	***0.000***	96.77	***0.001***	116.18	0.747	120.70	0.086	132.35	0.981	132.00	0.054
HDL Cholesterol (mg/dL). mean	41.65	0.249	45.99	0.125	42.27	0.836	38.61	0.503	40.48	0.839	39.69	0.432
Total cholesterol (mg/dL). mean	157.71	0.904	152.45	***0.004***	173.21	0.233	154.40	0.092	140.46	0.241	154.00	0.101
Triglycerides (mg/dL). mean	130.12	0.356	124.82	0.291	139.51	0.066	156.42	***0.000***	103.73	0.728	100.00	0.163
Diabetes (%)	53.43	***0.000***	13.69	***0.000***	39.20	***0.000***	65.34	0.455	71.42	0.728	66.66	***0.000***
Hypertension (%)	75.53	***0.039***	60.27	0.121	71.80	***0.022***	86.13	0.099	74.28	0.804	72.72	0.146
Metabolic syndrome (%)	46.99	***0.000***	13.69	***0.000***	47.13	0.364	54.44	0.478	47.61	0.147	27.27	0.296
FHRs (%). mean	29.02	0.709	25.69	0.271	23.28	***0.004***	35.81	0.412	31.90	***0.044***	49.47	***0.004***
FHRs (%) median (25–75 percentiles)	22.05 (8.98–42.75)	0.672	23.08 (3.46–41.62)	0.451	15.97 (3.61–34.95)	***0.001***	26.54 (13.98–57.09)	ns	30.55 (14.98–47.08)	***0.033***	51.46 (28.46–71.47)	***0.001***
FIB-4 mean	3.29	***0.018***	2.81	***0.000***	1.56	***0.000***	3.71	***0***.***020***	5.30	0.177	7.09	***0.000***
LSM kPa mean	13.21 (12.75)	***0.000***	5.08 (3.22)	0.838	7.76 (5.47)	***0.000***	13.629 (7.79)	***0.003***	25.23 (11.15)	***0.013***	54.07 (31.53)	***0.000***
PNPLA3 dominant model (CG + GG) (%)	57.93	***0.035***	38.35	0.242	50.66	***0.019***	71.28	0.448	62.85	0.718	51.51	0.122
MBOAT recessive model (TT) (%)	22.74	0.440	27.39	0.162	15.85	0.076	28.71	0.548	22.85	0.400	51.51	0.247
GCKR recessive model (TT) (%)	28.11	0.949	31.50	0.882	32.59	0.988	32.67	0.340	22.85	0.454	0	0.568
TM6SF2 dominant model (CT + TT) (%)	16.09	0.749	13.79	0.698	16.74	0.474	12.87	0.260	22.85	0.623	0	0.636

The text was in bold when *p* was considered statistically significant (ie <0.05)

An analysis of variance across the four groups reveals that age (*p* < 0.0001), AST (*p*: 0.002), diabetes prevalence (*p* < 0.0001), FHRs (*p*: 0.004), FIB-4 (*p* < 0.001) and LSM (*p* < 0.001) increase among classes.

Moreover, a univariate analysis was performed to correlate FHRs (as the dependent variable) with clinical and laboratory parameters of patients with MASLD. From this analysis, age (*p*: 0.001), male sex (*p*: 0.030), ALT (*p*: 0.015), glycaemia (*p*: 0.001), diabetes (*p* < 0.0001) HDL cholesterol (*p*: 0.011), hypertension (*p* < 0.0001), metabolic syndrome (*p* < 0.0001), FIB-4 (*p*: 0.001) and LSM (*p*: 0.005) were significantly associated with higher scores of FHR (Table 2). At the subsequent multiple linear regression analysis, including all the variables significant at the univariate, age (*p* < 0.0001), HDL cholesterol (*p*: 0.010) diabetes (*p* < 0.0001) and FIB-4 (*p*: 0.003) were confirmed independently associated with FHRs (Table 3a). However, because FHRs is a score calculated from age, sex, total cholesterol, HDL cholesterol and blood pressure (other than smoking habits), we also performed another multivariable analysis excluding age and HDL cholesterol with the aim of overcoming an eventual incorporation bias. In this calculation, only the presence of diabetes (*p* < 0.0001) and LSM (*p*: 0.033) remained correlated with FHRs (Table 3b).

**Table 2:** Univariate analysis vs FHRs as a dependent variable

Variable	*B*	95% CI	*p*
Age	**1.502**	**1.358–2.647**	**0.001**
Sex	**7.409**	**1.939–14.079**	**0.030**
AST	0.038	0.030–1.106	0.268
ALT	**2.079**	**1.015–3.142**	**0.015**
BMI	1.045	0.678–2.589	0.890
GGT	1.003	0.028–2.034	0.835
Glycaemia	**2.220**	**1.146–3.293**	**0.001**
HDL cholesterol	**0.292**	**0.067–0.517**	**0.011**
Total cholesterol	1.066	0.050–1.033	0.070
Triglycerides	1.011	0.330–1.056	0.610
Diabetes	**26.990**	**21.430–32.551**	**0.000**
Hypertension	**25.467**	**18.681–32.253**	**0.000**
Metabolic syndrome	**16.130**	**9.881–22.380**	**0.000**
FIB-4	**1.706**	**1.503–2.689**	**0.001**
LSM	**1.767**	**1.066–2.602**	**0.005**
PNPLA3 dominant model (CG + GG)	0.669	0.643–10.980	0.898
MBOAT recessive model (TT)	1.749	0.082–14.581	0.787
GCKR recessive model (TT)	1.972	0.382–12.326	0.706
TM6SF2 dominant model (CT + TT)	1.387	0.704–14.478	0.834

The text was in bold when *p* was considered statistically significant (ie <0.05)

**Table 3:** Multivariate analysis (multiple linear regression) vs FHRs as a dependent variable

(a) Coefficients							
Model	Unstandardized coefficients		Standardized coefficients	*t*	Sig.	95.0% confidence interval for *B*	
	*B*	Std. error	Beta			Lower bound	Upper bound
1							
(Constant)	− 42.596	9.749		− 4.369	0.000	− 62.022	− 23.170
Age	**1.118**	**0.099**	**0.725**	**11.297**	**0.000**	0.921	1.316
ALT	0.036	0.025	0.084	1.446	0.152	− 0.014	0.085
Sex	4.835	2.835	0.106	1.705	0.092	− 0.814	10.484
Glycaemia	− 0.041	0.046	− 0.061	− 0.876	0.384	− 0.133	0.052
HDL	**− 0.225**	**0.085**	**− 0.160**	**− 2.636**	**0.010**	− 0.395	− 0.055
Diabetes	**15.020**	**3.181**	**0.352**	**4.722**	**0.000**	8.681	21.358
Metabolic syndrome	4.868	2.582	0.118	1.885	0.063	− 0.277	10.013
FIB4	**1.628**	**0.535**	**− 0.224**	**− 3.045**	**0.003**	1.563	2.693
LSM	0.189	0.107	0.122	1.764	0.082	0.024	0.403

(b) Coefficients							
Model	Unstandardized coefficients		Standardized coefficients	*t*	Sig.	95.0% confidence interval for *B*	
	*B*	Std. error	Beta			Lower bound	Upper bound
1							
(Constant)	11.292	3.435		3.287	0.002	4.453	18.131
ALT	− 0.036	0.036	− 0.085	− 1.004	0.319	− 0.108	0.036
Diabetes	**24.239**	**4.270**	**0.569**	**5.677**	**0.000**	15.739	32.740
Metabolic syndrome	6.025	4.026	0.146	1.497	0.139	− 1.990	14.040
FIB4	1.593	0.818	0.031	0.724	0.041	1.036	2.221
LSM	**1.336**	**0.172**	**0.023**	**0.211**	**0.033**	1.037	2.380

The text was in bold when *p* was considered statistically significant (ie <0.05)

**Fig. 1:**
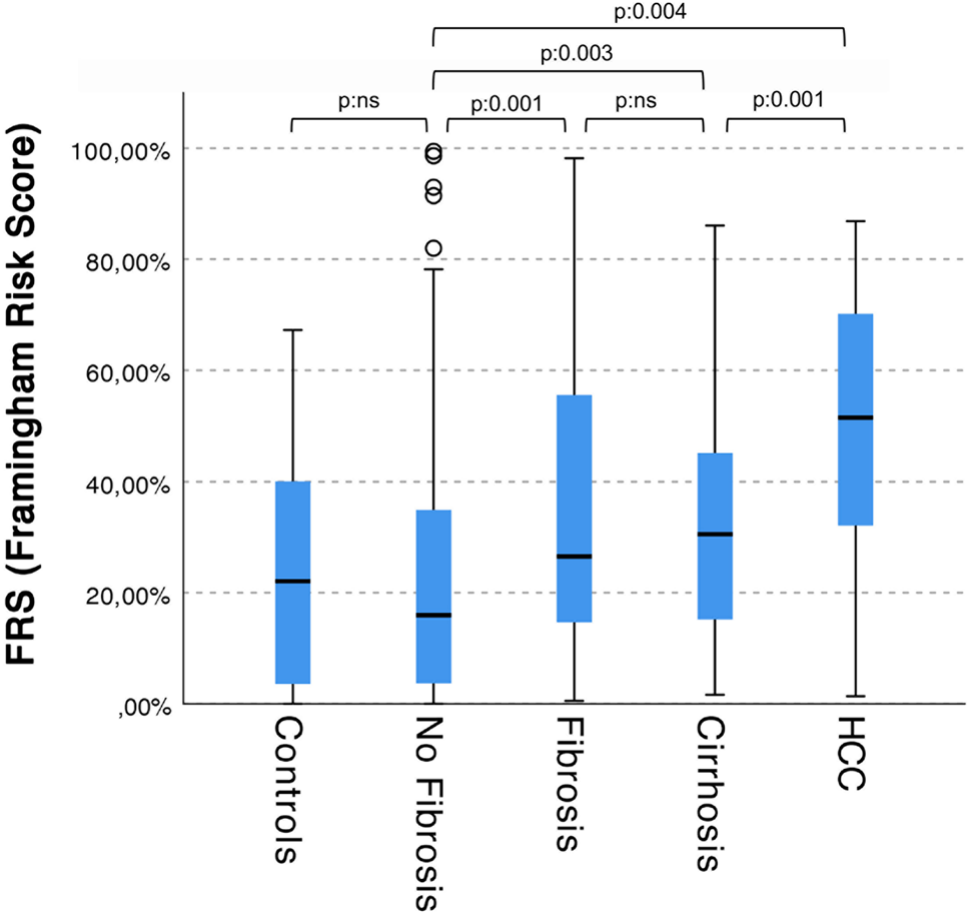
FHRs among patients’ classes. Group A: no fibrosis; group B: fibrosis; group C: cirrhosis; group D: HCC (*p* < 0.0001, independent samples Kruskal–Wallis test with 4 degrees of freedom; for the pairwise comparisons see the image)

Importantly, FHRs values significantly increased among the patients’ classes (*p* < 0.001, one-way ANOVA), as shown in Fig. 1.

### Endo-PAT subgroup analysis

In a subgroup of 110 patients (60.9% group A; 20.0% group B; 17.3% group C; 1.8% group D), we also assessed a peripheral artery tonometry measurement by EndoPAT, to evaluate if there was a measurable preclinical endothelial dysfunction. An altered EndoPAT (below 1.67) was found in 42 out of 110 subjects (38.2%). In particular, 31.8% of patients with low risk of fibrosis (group A) had endothelial dysfunction, 37.2% of group B (high risk of fibrosis), 52.6% of patients with clinical cirrhosis correlated to MASLD (group C) (p: ns). Group D was represented only by two subjects and, therefore, were excluded from further analyses. As expected, EndoPAT values correlated with FHRs (OR 19.078–2.584–35.571 95% CI—*p*: 0.024), demonstrating a direct correlation between the two parameters.

### Metabolomics analysis

Gas chromatography–mass spectrometry consistently detected 296 endogenous metabolites in each specimen. These compounds are involved in many biochemical processes, such as energy metabolism, lipid metabolism and amino acid metabolism. For chromatographic peak identification, the linear retention index difference max tolerance was set to 10, while the minimum matching for NIST library search of the corresponding mass spectrum was set to 85%. Results were summarized in a comma separated matrix file and loaded in the appropriate software for statistical manipulation. After data alignment using the parametric time wrapping algorithm [32] and peak picking, integration and deconvolution, the chromatographic data were tabulated with one sample per row and one variable (metabolite) per column. The normalization procedures consisted of data transformation and scaling. Data transformation was performed by generalized log transformation while data scaling was by auto scaling (mean-centered and divided by standard deviation of each variable) [35].

**Fig. 2:**
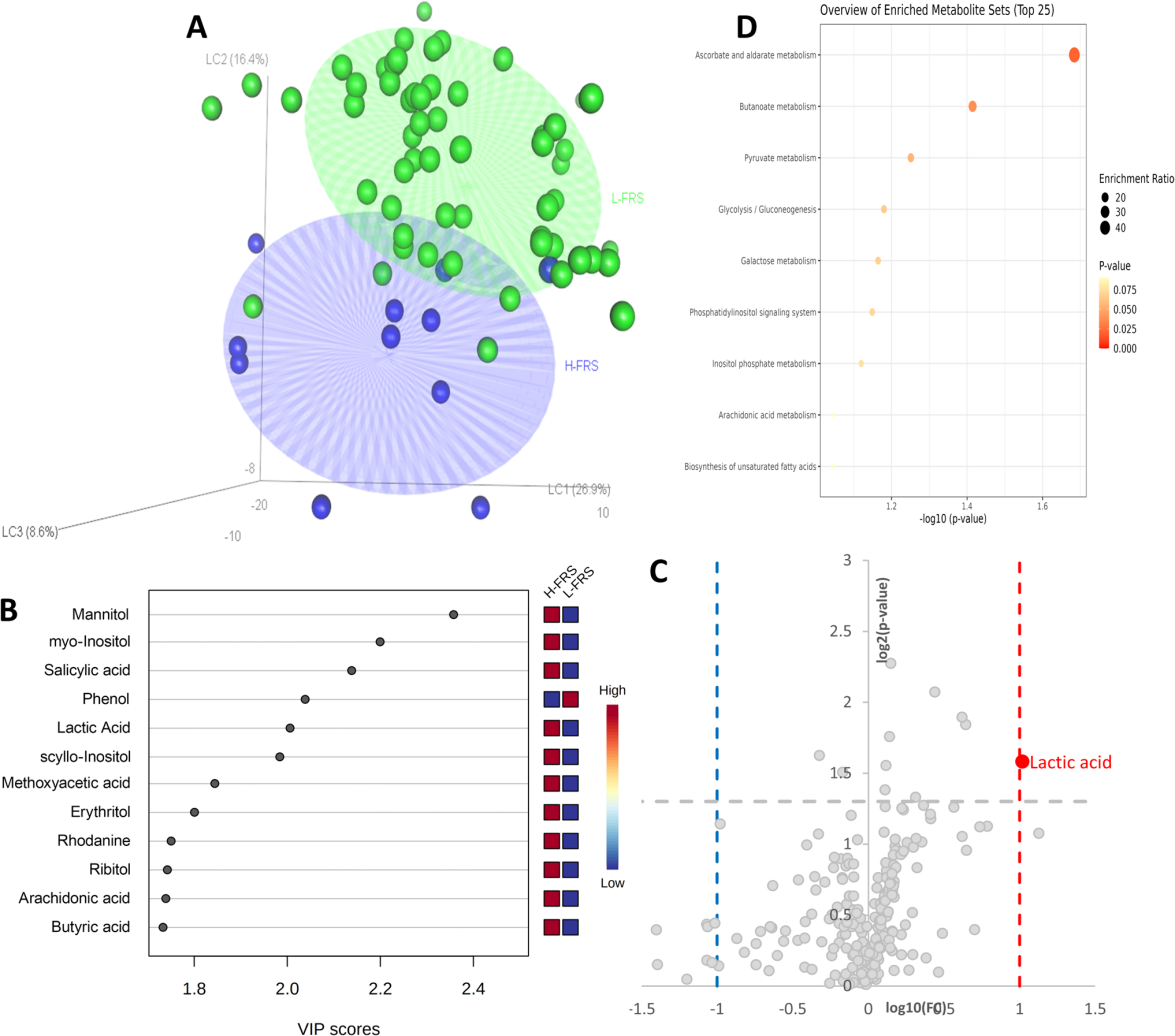
FHRs PLS-DA classification model: **a** high (H-FHRs > 40%, blue) vs low (L-FHRs < 40%, green) score plot, axes represent the latent components, the amount of explained variance were reported in bracket. **b** Metabolites showing a VIP score > 1.5 in the PLS‐DA analysis. **c** Volcano plot reporting metabolite concentration fold changes and their statistical significance comparing H-FHRs vs L-FHRs subjects. **d** Metabolite set enrichment analysis using the selected metabolites (VIP and Volcano) (Color figure online)

Metabolomics profiles were used to train several classification models based on PLS-DA algorithm. Model trained using at least 40% FHRs resulted significant (data not shown) while lower thresholds not, as shown in Fig. 2.

A well-defined differentiation of the high (H-FHRs) and low (L-FHRs) serum profiles was achieved (*R*^2^_Ycum_ = 0.89, *Q*^2^_Ycum_ = 0.57) (Fig. 2a), also showing a significant permutation test (*p* value = 0.007). Variable importance in projection (VIP) scores were calculated for each component in the PLS-DA regressions. Panel b of Fig. 2 shows the metabolites selected as being those most responsible for class separation (with a VIP-score > 1.5). Lactic acid was also selected by the volcano plot (Fig. 2c) showing both *p* value < 0.05 and a fold change (FC) higher than 2.0. All these metabolites were also analyzed in the context of a metabolites enrichment analysis resulting in the alteration of ascorbate and aldarate metabolism, butanoate metabolism, pyruvate metabolism and glycolysis/gluconeogenesis (Fig. 2d).

**Fig. 3:**
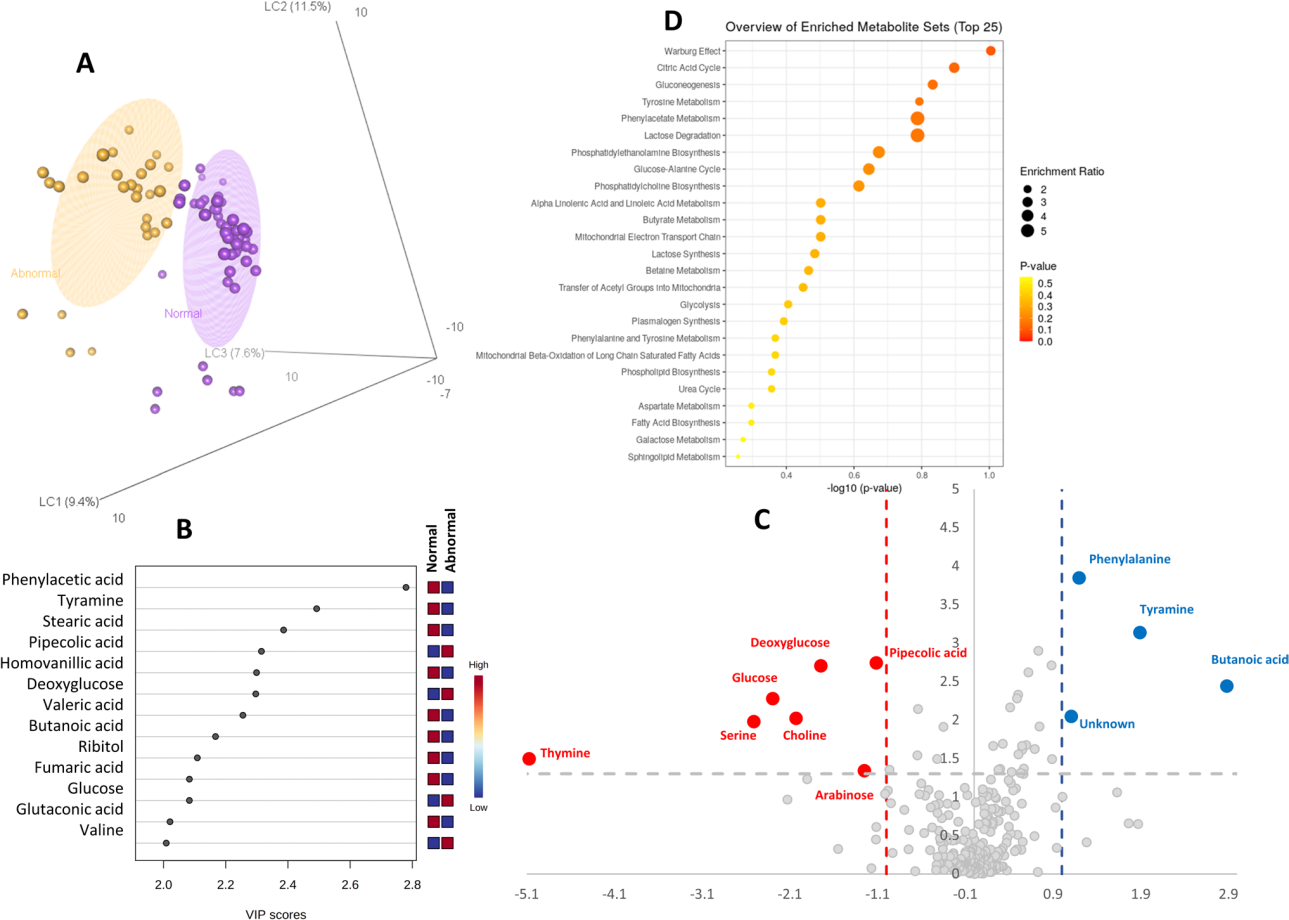
EndoPAT PLS-DA classification model: **a** abnormal (orange) vs normal (purple) score plot, axes represent the latent components, the amount of explained variance were reported in bracket. **b** Metabolites showing a VIP score > 1.5 in the PLS‐DA analysis. **c** Volcano plot reporting metabolite concentration fold changes and their statistical significance comparing normal vs abnormal EndoPAT. **d** Metabolite set enrichment analysis using the selected metabolites (VIP and Volcano) (Color figure online)

Good class separation was also achieved classifying samples on the basis of EndoPAT results (normal vs abnormal, *R*^2^_Ycum_ = 0.93, *Q*^2^_Ycum_ = 0.78) (Fig. 3a), also showing a significant permutation test (*p* value = 0.0035). variable importance in projection (VIP) scores were calculated for each component in the PLS-DA regressions. Panel b of Fig. 3 shows the metabolites selected as being those most responsible for class separation (with a VIP-score > 1.5). Phenylalanine, tyramine, butanoic acid and a metabolite for which was not possible to determine the structure resulted higher concentrated in patients with normal EndoPAT, while thymine, serine, glucose, choline, deoxyglucose, arabinose and pipecolic acid resulted with a higher concentration in patients with abnormal EndoPAT (Fig. 3c).

All these metabolites and the ones with a VIP-score higher than 1.5 were also analyzed in the context of a metabolites enrichment analysis resulting in the alteration of Warburg effect, Krebs cycle, gluconeogenesis, and tyrosine metabolism (Fig. 3d).

## Discussion

Nowadays, it is well known that CVD occurrence represents the most important factor of morbidity and mortality in patients with NAFLD/MASLD, in fact CVD events are higher than those liver-related [38]. Indeed, NAFLD/MASLD is accounted for being an independent CVD risk factor, even independent from smoke, obesity, diabetes, and metabolic syndrome (and its components, hypertension, dyslipidemia, visceral obesity and hyperglycemia) [39]. This peculiar characteristic is even more interesting today, as the pathogenetic relationship between non-alcoholic steatosis and the dysmetabolic state has been more forcefully established by the new definition of MASLD. A non-invasive assessment of the CVD risk for a MASLD patient should be the most appropriate approach, considering the global increasing burden of this condition, although this evaluation is always difficult. In the present study, we precisely aimed to evaluate the clinical risk of CVD occurrence by calculating it with a well-validated risk score (the 2008 version of the Framingham Hearth Risk score) and correlating it with a mostly non-invasive assessment (a precision medicine multi-OMIC approach) of MASLD severity. We believe that our interesting results clarify how and which MASLD patients should be attentioned for CVD risk. In fact, our overall MASLD cohort presents most of known CVD risk parameters (BMI, glycemia, prevalence of hypertension, diabetes and metabolic syndrome) significantly different, apart from those correlated with the liver disease itself (such as FIB-4 and LSM), but not FHRs itself, compared to age and sex-matched controls. At a first glance, these results look inconsistent with the supposed higher CVD risk in NAFLD patients, however, further analyzing the data we found out that only patients with low risk of advanced fibrosis (group A) and controls had similar FHRs. In fact, FHR scores were significantly different between controls and MASLD patients of group B, C and D (aggregated) (*p*: 0.024, Mann–Whitney *U* for independent samples), whereas there was no statistical difference between controls and group A (*p*: 0.451). Indeed, the group A patients had very likely simple steatosis, a metabolic condition very similar to matched healthy controls. Therefore, our finding seems to confirm, once again, that simple steatosis doesn’t confer any additional risk to its carrier.

Interestingly, analyzing FHRs among the four groups of MASLD, we observed a statistically significant increasing trend (as showed in Table 1 and Fig. 1; *p*: 0.004). These findings suggest that the non-invasive algorithm used to stratify MASLD patients based on fibrosis, was useful also to predict their CVD risk. In turn, this confirms previous reports that one of the strongest predictors of CVD risk in MASLD is the severity of the liver disease itself [40]. However, to better understand the relationship between CVD risk and MASLD disease we performed univariate and multivariate analyses with FHRs as the dependent variable. In the univariate analysis (Table 2), we observed well knows CVD risk factors associated with higher values of FHRs, such as age, sex and metabolic syndrome (and its components: glycaemia, hypertension, HDL cholesterol) and diabetes, apart from factors associated with liver disease (ALT, FIB-4 and LSM). Interestingly, the SNPs for NAFLD/MASLD risk were not correlated with FHRs. At the multivariate analysis, performed with all the significant variables at the univariate as independent factors, age, HDL, diabetes and FIB-4 were confirmed independently associated with FHRs (Table 3a). However, since FHRs is calculated from age, sex, total cholesterol, HDL cholesterol and blood pressure (other than smoking habits), there is a high risk of an incorporation bias on the statistical significance of these variables. Therefore, we performed another multivariate analysis, excluding Age and HDL cholesterol (Table 3b). Thus, only diabetes and LSM were significantly associated with higher FHRs, demonstrating, again, that liver disease severity (also if assessed in a non-invasive way) is the stronger driver of CVD risk, together with the presence of type 2 diabetes mellitus.

FHRs has the limitation to be precisely a risk score, calculated from clinical parameters, therefore there is no direct measurement of any pathophysiological impairment predisposing the patient to CVD. For this reason, we also performed, in a subgroup of patients, a direct measurement of endothelial dysfunction, which is the early vascular derangement that correlates with CVD [41]. Endothelial dysfunction, measured by peripheral artery tonometry was already present in 38.2% of the 110 patients who underwent the procedure. Overall, there was no differences between the classes of fibrosis risk, however there was a higher percentage (52.6% vs 39.3%) of altered endoPAT in group C (clinical cirrhosis) compared to all the other groups that did not reach the statistical significance due to the small sample size. Moreover, we found a linear correlation between endoPAT values and FHRs, as expected. Since the included patients were all free from active/previous CVD, these findings suggest that an already existing vascular derangement is, once again, correlated with the worst-case scenarios concerning liver disease severity.

Noteworthy, several interesting results emerged from the untargeted metabolomics analyses. First, the PLS-DA analysis, conducted to discover metabolic difference in FHRs, revealed no differences in metabolites profiles below the value of 40%. This is of particular significance, because it is reported that a FHRs > 30% is classified as high CVD risk. In our cohort, the number of patients with FHRs ≥ 40% was 65 out of 466 (13.95%), 21 out of 185 (11.35%) in group A, 18 out of 83 in group B (21.68%), 18 out of 86 in group C (20.93%) and 5 out of 26 in group D (19.24%). Also, 10 out of 73 controls (13.69%) had this FHR score. Once again, there was a statistically significant difference between group A and the other groups (A vs B *p*: 0.027; A vs C *p*: 0.037, Mantel–Haenszel), and no difference with controls (*p*: 0.602). Very interestingly, the metabolites classified as predicting an FHRs > 40% were in the ascorbate and aldarate metabolism, butanoate metabolism, pyruvate metabolism and glycolysis/gluconeogenesis. Ascorbate (vitamin C) is a well-known anti-oxidant, associated with CVD and cancer protection [42]. Butanoate metabolism derangement showed detrimental effects on CVD risk and endothelial dysfunction [43, 44]. In fact, it is well known that the decrease of butyrate-producing bacteria abundance in the Gut-microbiota leads to increased CVD risk [45]. Pyruvate metabolism impairment has been associated with increased fatty acid oxidation, mitochondrial dysfunction (typical of NAFLD/NASH), and reduced cardiac efficiency [46]. Finally, also glycolysis/gluconeogenesis intermediates have been demonstrated to contribute to metabolic classes separation between subjects with a high (> 40%) and a low FHRs. This finding is unsurprising, given the well-known association between diabetes/insulin resistance and CVD risk.

The metabolomics analysis on endothelial dysfunction, measured by EndoPAT, revealed an association between higher concentrations of butanoate, phenylalanine, and tyramine and normal EndoPAT values. In accordance with the forementioned supposed protective mechanisms against CVD, Butanoate concentrations are higher in patients without ED [43–45]. Also, higher phenylalanine levels in patients without preclinical ED is in line with previous reports which highlight that its concentrations are inversely correlated with carotid atherosclerosis and CVD risk [47]. As well, low levels of tyramine have been associated with cardiometabolic risk and inflammation in Metabolic Syndrome, therefore, finding it elevated in normal EndoPAT has a plausible explanation [48]. Conversely, thymine, serine, glucose, choline, deoxyglucose, arabinose and pipecolic acid were in higher concentrations in patients with ED (abnormal EndoPAT). Thymine was already demonstrated as a marker of coronary artery disease (CAD) in one metabolomic study [49]. Unsurprisingly, we observed high levels of glucose and deoxyglucose in patients with ED, in accordance with the already known association between hyperglycemic states and CVD risk. As well, high levels of Choline have been associated with major cardiovascular events in patients with myocardial infarction [50]. Pipecolic acid is an intermediate metabolite of the essential amino acid lysine whose degradation metabolites have been associated with the risk of type 2 diabetes and cardiovascular disease [51]. On the contrary, arabinose, a dietary pentose, has been associated with protective effects on diabetes, metabolic syndrome and dyslipidemia [52]. In the same way, high levels of the amino acid Serine have been inversely associated with the risk of peripheral artery disease in previous studies [53]. Therefore, our contradictory findings on these two metabolites need more explanations in future studies; perhaps, however, we could hypothesize a “metabolic attempt” to compensate the endothelial dysfunction in a preclinical stage.

Finally, it has to be noticed that in our cohort, the most studied SNPs for NAFLD/MASLD risk didn’t intercept any adjunctive cardiovascular risk. This could be mostly due to several reasons. First, these SNPs were identified mostly by inferring from the liver disease severity (inflammation and fibrosis), and most of them have specific activity in the processes of inflammation and fibrosis within the liver. Furthermore, even if some of them (i.e. *TM6SF2*) have been reported to exert also an effect on cardiometabolic risk (both detrimental and protective), it is not known already their penetrance in the general population, apart from NAFLD patients. Finally, our patients showed no statistically different SNPs prevalence among the liver disease severity groups, except for PNPLA3 dominant model that was more represented (as expected) in MASLD overall compared to controls, and in patients with higher risk of fibrosis (Group B) compared to those with lower risk (group A). Therefore, a possible explanation is that these SNPs are to be taken into account only partially for liver disease severity which, in turn, is itself the major marker for CVD risk.

However, a convincing theory postulated that “Metabolic NAFLD” could be different from “Genetic NAFLD” [54], therefore, we performed such an analysis in our population. Thus, we clustered MASLD patients in “Genetic” (subjects having at least one pathological mutation in any of the SNPs we analyzed) and “Non-genetic” (no mutations at all) MASLD. One more time we found that patients with “Genetic MASLD” had an increased risk of liver fibrosis, but not an adjunctive CVD risk (see supplementary Table 2).

Taken as a whole, we believe that our approach, if confirmed and validated in larger cohorts and in other populations, could be a valid strategy to correctly address the clinical risk of MASLD patients, both in terms of what type (e.g. CVD and/or liver-related) and what degree. Our method could possibly represent a cost-effective diagnostic tool for such a large population, which in turn may have an impactful effect in decreasing the costs of National Healthcare systems, both by improving disease progression prevention and diagnosis.

Finally, our present work has some limitations to discuss. First of all, the present data are to be considered cross-sectional. Therefore, no data are available at the time, on the real incidence of CV diseases in our population. However, the score we chose (FHR) has been widely demonstrated to be reliable in predicting CVD risk in longitudinal studies. Moreover, by study protocol, we didn’t collect liver biopsy of every single patient in the cohort, but only in 68 of them (14.59%). Therefore, it is likely that there is no absolute certainty on liver disease severity in each patient. However, our data showed a good discrimination between the classes in terms of both clinical and omics data, thus successfully demonstrating the effectiveness of this diagnostic algorithm, which is also recommended by guidelines. Furthermore, the effective presence of endothelial dysfunction by EndoPAT was measured only in a subgroup of patients. However, our data demonstrated that there was a significant linear correlation between PAT results and FHR score. Finally, it has to be mentioned that our study population had an average age of about 65 years (65.35 years for controls and 66.67 years for MASLD patients), therefore, it is to be considered a high CVD risk category, as universally agreed [55]. Therefore, even if our data demonstrated that, when accounting for the incorporation bias of the FHRs calculation, the presence of T2DM and higher liver stiffness values were the only variables that intercepted the higher CVD risk, it could be advisable to repeat such analyses in further longitudinal studies in younger populations to verify our findings in lower risk settings.

## Conclusions

Our data demonstrated that: (A) a noninvasive diagnostic approach based on clinical, laboratory, and OMICs data is capable of identifying the severity of liver disease and the CVD risk of MASLD patients in a reliable way. (B) CVD risk is mostly correlated to MASLD disease severity, increasing together with it. (C) Metabolomic profiles of high CVD risk patients showed peculiar metabolic pathways involved that could be considered as therapeutic targets. Further longitudinal studies should be necessary to verify the occurrence of CVD events in such population.

## Supplementary Information

Below is the link to the electronic supplementary material.

Supplementary file1 (DOCX 48 KB)

## Data Availability

The data that support the findings of this study are available from the corresponding author upon reasonable request.

## Abbreviations

CVD: Cardiovascular disease
FHRs: Framingham Heart Risk Score
MASLD: Metabolic-associated steatotic liver disease
T2DM: Type 2 diabetes mellitus
FIB-4: Fibrosis-4 score
NFS: NAFLD fibrosis score
LSM: Liver stiffness measurement
SNP: Single nucleotide polymorphism
GC–MS: Gas chromatography–mass spectrometry
PAT: Peripheral arterial tonometry
PLS-DA: Partial least square discriminant analysis
VIP: Variable importance in projection
ALT: Alanine aminotransferase
AST: Aspartate amino-transferase
BMI: Body mass index
