# Supplementary material for: Evaluating cardiovascular risk in metabolic steatosis with precision medicine non-invasive approaches: insights from a cohort study
Source: Intern Emerg Med. 2024 May 16;19(8):2293–307. doi: 10.1007/s11739-024-03626-3 (PMC11582347; doi:10.1007/s11739-024-03626-3)
Supplement: Supplementary file 1 — Supplementary file1 (DOCX 48 KB) [file 11739_2024_3626_MOESM1_ESM.docx]

**Supplementary Data**

**Title:** Evaluating Cardiovascular Risk in Metabolic Steatosis with Non-Invasive approaches: Insights from a Cohort Study

**Authors:** Mario Masarone MD PhD^1^*, Benedetta M. Motta PhD^1^*, Pietro Torre MD^1^, Marco Aquino MD^1^, Federica Belladonna MD^1^, Martina Lombardi^2^, Jacopo Troisi^1,2^, Marcello Persico MD^1^

1: Department of Medicine, Surgery and Dentistry, “Scuola Medica Salernitana”, University of Salerno, Baronissi, SA, Italy

2: Theoreo srl, Montecorvino Pugliano, SA, Italy

*these authors equally contributed to the present work

**Journal:** Internal and Emergency Medicine

**SUPPLEMENTAL CONTENTS CAPTIONS**

**Supplementary Data:**

- Clinical evaluation of subjects included in the study.
- Genetic analysis.
- Metabolomics analysis chemical and statistical methods.
- Supplementary table 1 and 2

**Patients and Methods**

**Clinical evaluation**

For each subject we recorded: clinical history with alcohol consumption, previous/current cardiovascular diseases (hypertension, angina, STEMI, N-STEMI, stroke etc.) and smoking habits, physical examination with arterial pressure measurement, waist circumference, body mass index (BMI), blood glucose, Homeostasis Model of Assessment for Insulin Resistance (HOMA), total and fractioned cholesterol, triglycerides, AST, ALT, GGT, ALP, complete blood count, metabolic syndrome diagnosis by NCEP-ATPIII criteria^1^. Also, a complete drug history, with particular regard to CVD medications, was recorded.

*Liver disease assessment*: An abdomen ultrasound examination with the evaluation of the liver echo pattern of liver steatosis was performed by a skilled ultrasonographist at the time of enrollment^2^. After the assessment of the presence of steatosis, in absence of any other liver disease (viral, alcohol, autoimmune, metabolic other than NAFLD/MASLD), the patient was evaluated on the basis of the diagnostic protocol proposed by the Italian Guidelines on NAFLD diagnosis and management^3^. With this purpose AST to Platelet Ratio Index (APRI), NFS and FIB-4 scores were evaluated for every patient^4-6^. Liver Stiffness measurement (LSM) with vibration controlled transient elastography - VCTE (FibroScan® Mini+ 430; EchoSense, Paris, France; equipped with an M or an XL probe) were performed by a skilled hepatologist (MP and MM). LSM were considered reliable, and reported, only if there was an interquartile range (IQR) < 30% of the mean value and a success rate of at least 60% of the measurements. Patients were defined as having cirrhosis when the typical clinical/ultrasonographical signs (esophageal varices, spleen enlargement, low platelet count, caudate lobe hypertrophy at ultrasound examination) were detected. Based on these evaluations, and the clinical assessment, they were then stratified in three categories: low risk of advanced fibrosis (Group A), high risk of advanced fibrosis (Group B), clinical cirrhosis (Group C). Those at uncertain risk of fibrosis (defined as those individuals in whom LSM measurement was unsuccessful/not reliable and there were no indications of clinical cirrhosis) underwent to liver biopsy. In the present study, 68 (14.59%) patients underwent to liver biopsy by this diagnostic algorithm (Supplementary data). A group of patients with a diagnosis of steatosis-associated hepatocellular carcinoma were also included (group D). The diagnosis of NAFLD-associated HCC was made when the EASL diagnostic criteria for HCC were met in a NAFLD patient^7^.

**Framingham Heart Risk Score Calculation**

Total cholesterol levels >200 mg/dL (≥5.2 mmol/dl), LDL cholesterol >130 mg/dL (>3.3 mmol/dl), HDL cholesterol <40 mg/dL (<1.03 mmol/dl), and triglycerides 150 mg/dL (≥1.7 mmol/dl), were considered as risks for the FHR score calculation. A CVD risk percentage at 10 years was classified as low risk if FHR was <10%, intermediate risk if 10%–30%, and high risk if >30%^8-10^.

**Serum and Plasma collection**

Serum samples were collected from a peripheral vein in 5 mL sterile vacutainers containing gel separator and clot activator. Each sample was allowed to clot at room temperature and then was centrifuged at 1000g for 20 min. Serum aliquots were recovered, transferred into prelabeled cryovials, and stored at −80 °C. EDTA plasma samples were collected from a peripheral vein in two 2 mL sterile vacutainers containing tri-potassium ethylenediaminetetraacetate (K3EDTA). Each plasma sample was centrifuged at 1000g for 20 min and then plasma aliquots were recovered, transferred into prelabeled cryovials, and stored at −80 °C.

**Genetic Study**

**SNPs Genotyping** DNAs have been extracted from peripheral blood using QIAamp DNA Blood Mini Kit (QIAGEN Inc., MD, USA) and quantified by NanoPhotometer® NP80 (Implen GmbH, München, Germany). The single‐nucleotide polymorphisms (SNP) rs738409 (I148M, *PNPLA3*), rs58542926 (E167K, *TM6SF2*), rs641738 (*MBOAT7*), and rs1260326 (P446L, *GCKR*), previously associated with the susceptibility to NAFLD, have been genotyped in duplicate by TaqMan 5’-nuclease allelic discrimination assays (Applied Biosystems by Thermo Fisher Scientific). No transcript controls (NTC) and Minor-allele positive controls were distributed across plates for concordance checking.

Post-PCR allelic discrimination was performed measuring allele-specific fluorescence on LightCycler® 480 System (Roche Diagnostics, IN, USA). Genotype frequencies were assessed for Hardy–Weinberg equilibrium using the goodness-of-fit χ2 test.

The contribution of genetic factors was estimated by assuming an additive, dominant or recessive genetic model, separately. The PNPLA3 I148M variant was coded in an additive model^11^. TM6SF2 E172K variant was coded in a dominant genetic model because of its relatively low allele frequency. The MBOAT7 rs641738 C>T variant impacts the severity of NAFLD through a recessive model of inheritance^12^. The GCKR rs1260326 C>T fitted better with a recessive model of inheritance^13^.

**Metabolomics Analysis**

GC-MS analyses were performed on a two-microliter sample of the derivatized solution. Chromatographic separation was achieved with a 30 m 0.25 mm CP-Sil 8 CB fused silica capillary GC column with 1.00 µm film thickness from Agilent (Agilent, J&W), with helium as carrier gas.

The initial oven temperature of 100 °C was held for 1 min and then raised to 320 °C at a rate of 6 °C/min, with further 2.33 minutes of holding time. The gas flow was set to achieve a constant linear velocity of 39 cm/s and the split flow was set to 1:2. The mass spectrometer was operated in electron impact (70 eV) in full scan mode in the interval of 35-600 m/z with a scan velocity of 3333 amu/sec and a solvent cut time of 5 minute. The complete GC program duration was 40 minutes.

**Statistical analysis**

**Metabolomics data**

***Feature selection***

In genetic algorithms for feature selection, “mutation” means switching features on and off and “crossover” means interchanging used features. Feature selection was performed by means of the “Optimize Selection (Evolutionary)” algorithm implemented in Rapid Miner Studio ver. 9.10.011 (RapidMiner GmbH, Boston, MA, USA)^14^. These features were used to train the classification models.

***Partial Least Square Discriminant Analysis (PLS-DA)***

PLS-DA is a supervised method that uses multivariate regression techniques to extract, by means of linear combinations of original variables, the information able to predict class membership. PLS regression was performed by means of the MetaboanalystR^15^ package that uses the plsr function from the R pls package^16^. Classification and cross-validation were performed using the wrapper function from the caret package^17^.

***Identification of relevant metabolites***

The molecular identity of the metabolites of interest (i.e., metabolites with a VIP-score >2.0^18^, or in the areas of interest in volcano plot diagram) was determined by comparing the corresponding mass spectrum with a mass spectrum library^19^. These identified metabolites were further confirmed using external standards, according to the level 1 Metabolomics Standards Initiative (MSI)^20^.

Metabolic pathways involvement was evaluated using the MetPa tool^21^. It is based on the over-representation analysis tests. If a particular group of compounds is represented more than expected by chance within the compound list this pathway is selected. The over-representation was based on Fishers’ Exact test.

**References:**

1. Expert Panel on Detection E, Treatment of High Blood Cholesterol in A. Executive Summary of the Third Report of the National Cholesterol Education Program (NCEP) Expert Panel on Detection, Evaluation, and Treatment of High Blood Cholesterol in Adults (Adult Treatment Panel III). *JAMA: The Journal of the American Medical Association*. 2001;285(19):2486-2497. doi:10.1001/jama.285.19.2486

2. Palmentieri B, Desio I, Lamura V, et al. The role of bright liver echo pattern on ultrasound B-mode examination in the diagnosis of liver steatosis. *Digestive and Liver Disease*. 2006;38(7):485-489. doi:10.1016/j.dld.2006.03.021

3. Marchesini G, Bugianesi E, Burra P, et al. Non-alcoholic fatty liver disease in adults 2021: A clinical practice guideline of the Italian Association for the Study of the Liver (AISF), the Italian Society of Diabetology (SID) and the Italian Society of Obesity (SIO). *Digestive and Liver Disease*. 2022;54(2):170-182. doi:10.1016/j.dld.2021.04.029

4. Wai C. A simple noninvasive index can predict both significant fibrosis and cirrhosis in patients with chronic hepatitis C. *Hepatology*. 2003;38(2):518-526. doi:10.1053/jhep.2003.50346

5. Angulo P, Hui JM, Marchesini G, et al. The NAFLD fibrosis score: A noninvasive system that identifies liver fibrosis in patients with NAFLD. *Hepatology*. 2007;45(4):846-854. doi:10.1002/hep.21496

6. Sterling RK, Lissen E, Clumeck N, et al. Development of a simple noninvasive index to predict significant fibrosis in patients with HIV/HCV coinfection. *Hepatology*. 2006;43(6):1317-1325. doi:10.1002/hep.21178

7. Liver. EAftSot. EASL Clinical Practice Guidelines: Management of hepatocellular carcinoma. *J Hepatol*. Jul 2018;69(1):182-236. doi:10.1016/j.jhep.2018.03.019

8. Perk J, De Backer G, Gohlke H, et al. European Guidelines on cardiovascular disease prevention in clinical practice (version 2012). The Fifth Joint Task Force of the European Society of Cardiology and Other Societies on Cardiovascular Disease Prevention in Clinical Practice (constituted by representatives of nine societies and by invited experts). *Eur Heart J*. Jul 2012;33(13):1635-701. doi:10.1093/eurheartj/ehs092

9. WHO. HEARTS - Technical package for cardiovascular disease management in primary

health care: Risk-based CVD management. pdf. Accessed Jan 11, 2023. 2023.

10. Ford ES, Giles WH, Mokdad AH. The distribution of 10-Year risk for coronary heart disease among US adults: findings from the National Health and Nutrition Examination Survey III. *J Am Coll Cardiol*. May 19 2004;43(10):1791-6. doi:10.1016/j.jacc.2003.11.061

11. Sookoian S, Pirola CJ. Meta‐analysis of the influence of I148M variant of patatin‐like phospholipase domain containing 3 gene (PNPLA3) on the susceptibility and histological severity of nonalcoholic fatty liver disease. *Hepatology*. 2011;53(6):1883-1894. doi:10.1002/hep.24283

12. Teo K, Abeysekera KWM, Adams L, et al. rs641738C>T near MBOAT7 is associated with liver fat, ALT and fibrosis in NAFLD: A meta-analysis. *Journal of Hepatology*. 2021;74(1):20-30. doi:10.1016/j.jhep.2020.08.027

13. Di Costanzo A, Belardinilli F, Bailetti D, et al. Evaluation of Polygenic Determinants of Non-Alcoholic Fatty Liver Disease (NAFLD) By a Candidate Genes Resequencing Strategy. *Scientific Reports*. 2018;8(1)doi:10.1038/s41598-018-21939-0

14. Jungermann F. Information Extraction with Rapidminer. In: Hoeppner W, ed. *Proceedings of the GSCL Symposium ’Sprachtechnologie und eHumanities*. Universit ̈at Duisburg-Essen, Abteilung fu ̈r Informatik und Angewandte Kognitionswissenschaft Fakult ̈at fu ̈r Ingenieurwissenschaften; 2009: 50–61.

15. Chong J, Yamamoto M, Xia J. MetaboAnalystR 2.0: From Raw Spectra to Biological Insights. *Metabolites*. Mar 22 2019;9(3)doi:10.3390/metabo9030057

16. Mevik B-H, Wehrens R. The pls Package: Principal Component and Partial Least Squares Regression in R. *Journal of Statistical Software*. 2007;18(2)doi:10.18637/jss.v018.i02

17. Kuhn M. Building Predictive Models in R Using the caret Package. *Journal of Statistical Software*. 2008;28(5)doi:10.18637/jss.v028.i05

18. Akarachantachote N, Chadcham S, Saithanu K. Cutoff Threshold of Variable Importance in Projection for Variable Selection. *International Journal of Pure and Apllied Mathematics*. 2014;94(3)doi:10.12732/ijpam.v94i3.2

19. Stein S. Mass Spectral Reference Libraries: An Ever-Expanding Resource for Chemical Identification. *Analytical Chemistry*. 2012;84(17):7274-7282. doi:10.1021/ac301205z

20. Sumner LW, Amberg A, Barrett D, et al. Proposed minimum reporting standards for chemical analysis. *Metabolomics*. 2007;3(3):211-221. doi:10.1007/s11306-007-0082-2

21. Xia J, Wishart DS. MetPA: a web-based metabolomics tool for pathway analysis and visualization. *Bioinformatics*. 2010;26(18):2342-2344. doi:10.1093/bioinformatics/btq418

|  | **overall** |  | **controls** |  | **MASLD (466)** | | | | | | | ***p***  overall |
| --- | --- | --- | --- | --- | --- | --- | --- | --- | --- | --- | --- | --- |
|  |  | **<p>** |  | **<p>** | **Group A** | **<p>** | **Group B** | **<p>** | **Group C** | **<p>** | **Group D** |  |
| **Hypertension drugs (%)** | 69.09 | ***.000*** | 41.09 | ***.000*** | 66.07 | ***.019*** | 79.20 | *.****043*** | 66.67 | 1.000 | 66.67 | .103 |
| **Lipid lowering agents (%)** | 13.51 | .053 | 5,47 | .089 | 13.21 | .932 | 12.87 | .767 | 14.28 | **.**902 | 15.15 | .980 |
| **Oral antidiabetics (%)** | 26.60 | ***.000*** | 2.73 | ***.000*** | 29.07 | .312 | 34.65 | ***.045*** | 21.90 | ***.001*** | 0 | ***.022*** |
| **Insulin (%)** | 22.53 | ***.000*** | 1.37 | ***.000*** | 12.33 | .299 | 16.83 | .088 | 26.66 | ***.001*** | 60.60 | ***.000*** |

**Supplementary Table 1:** Drugs use prevalence among study population. As expected, Hypertension Drugs, Lipid Lowering agents and Antidiabetic drugs use was significantly higher in the MASLD group in respect to healthy controls. Other marginal differences were: a slightly higher use of antihypertensive drugs in group B as a consequence of the slightly higher prevalence of hypertension in this group and the differences among the groups about the use of oral antidiabetic or insulin (the latter more used in the more advanced groups).

| **Variable** | **Genetic MASLD** | **Non-Genetic MASLD** | **Univariate**  **p** | **Multivariable**  **p** |
| --- | --- | --- | --- | --- |
| **Age (Years)**  Mean ± SD | 67.21 ± 13.87 | 66.47 ± 15.17 | 0.825 | - |
| **Male Sex**  (%) | 64.1 | 58.1 | 0.250 | - |
| **BMI (kg/m^2^)**  Mean ± SD | **32.35 ± 6.59** | **30.58 ±5.78** | **0.043** | 0.487 |
| **AST (U/L)**  Mean ± SD | 38.64 ± 36.69 | 46.81 ± 52.03 | 0.184 | - |
| **ALT (U/L)**  Mean ± SD | 40.68 ± 36.69 | 46.47 ± 52.09 | 0.303 | - |
| **GGT (U/L)**  Mean ± SD | 86.88 ± 95.53 | 91.81 ± 112.82 | 0.823 | - |
| **Glycaemia (mg/dL)**  Mean ± SD | 118.54 ±42.184 | 122.48 ± 41.48 | 0.269 | - |
| **HDL Cholesterol (mg/dL)**  Mean ± SD | 39.32 ± 14.69 | 41.96 ± 13.92 | 0.90 | - |
| **Total Cholesterol (mg/dL)**  Mean ± SD | 159.05 ± 46.89 | 159.05 ± 46.02 | 0.879 | - |
| **Triglycerides (mg/dL)**  Mean ± SD | 133.29 ± 78.65 | 130.19 ± 72.01 | 0.708 | - |
| **Diabetes**  (%) | 58.0 | 48.7 | 0.112 | - |
| **Hypertension**  (%) | **83.2** | **69.8** | **0.009** | 0.530 |
| **Metabolic Syndrome**  (%) | 52.3 | 43.1 | 0.109 | - |
| **FHRs (%)**  Mean± SD | 31.85 ± 25.30 | 27.45 ± 24.64 | 0.146 | - |
| **FIB-4**  Mean ± SD | **3.90 ± 3.07** | **2.71 ± 2.27** | **0.002** | 0.487 |
| **LSM (kPa)**  Mean ± SD | **15.69 ± 14.91** | **9.422 ± 6.10** | **0.001** | 0.063 |

**Supplementary Table 2**

Univariate (Non-parametric Independent-Samples Mann-Whitney U Test for continuous variables, Pearson Chi-Square for discrete variables) and binary logistic multiple regression analysis comparing “Genetic” and “Non-Genetic” MASLD. Patients with MASLD were classified of having a “Genetic MASLD” if they had at least one mutation at risk of any of the 4 SNPs analyzed (PNPLA3, MBOAT, TM6SF2, GCKR). As it can be seen, patients with genetic MASLD had higher BMI, higher prevalence of hypertension, and higher likelihood of fibrosis (significantly higher FIB-4 and LSM). However, those differences weren’t independent predictors of “Genetic MAFLD (dependent variable) on the binary logistic multiple regression.
